# Study on the mechanism of multidimensional cutting teeth and the influencing factors of rock breaking efficiency

**DOI:** 10.1371/journal.pone.0297176

**Published:** 2024-03-08

**Authors:** Lin Chen, Debo Li, Jingbin He, Leifeng Meng, Qifu Chi, Gang Li, Weilin Chen, Ying Zhao, Xianzhong Yi, Chengyu Xia

**Affiliations:** 1 Yangtze University, Jingzhou, China; 2 CCDC ChangQing General Drilling Company, China; 3 China Petroleum West Drilling Engineering Co., LTD Downhole Operation Company, China; Bavarian Environment Agency, GERMANY

## Abstract

The innovative cutting mechanism of multi-dimensional teeth presents a groundbreaking approach to drill bit design, particularly optimizing drilling efficiency in challenging geological formations such as interlayers and gravel-rich layers within the Changqing Oilfield. Nevertheless, compared to conventional flat-tooth PDC drill bits, several aspects of the cutting mechanism and design parameters for multi-dimensional teeth require further elucidation. This article employs a linear cutting finite element model to establish cutting models for traditional flat teeth and two distinct types of multi-dimensional teeth, designated as Ridge and Benz. It systematically investigates the influence of varying cutting parameters on the effectiveness of rock-crushing within the multi-dimensional tooth-cutting mechanism. This study conducts laboratory-based single-tooth rock-crushing experiments to validate the numerical simulation results. Furthermore, applying principles derived from soil plastic mechanics contrasts the stress states experienced by rocks during the rock-crushing process between multi-dimensional teeth and conventional flat teeth, shedding light on the rock-crushing mechanism employed by multi-dimensional teeth. This research categorizes PDC cutting teeth on the drill bit into two groups: those near the center and those near the outer shoulder. A linear cutting model for teeth positioned near the outer shoulder is developed to analyze the impacts of different rake angles, side clearance angles, and welding errors on the tooth helix angle and the rock-crushing efficiency of the Benz tooth. This comprehensive study is a valuable reference for tailored drill bit design and holds potential for publication in a prestigious scientific journal.

## Introduction

As the development of shale gas, tight oil and gas, deep and ultra-deep wells and gas, and deepwater oil and gas resources intensifies, a growing trend toward larger-scale drilling operations in challenging formations exists. These formations are characterized by their hardness, abrasiveness, and heterogeneity. Foreign drill bit manufacturers have extensively researched Polycrystalline Diamond Compact (PDC) cutting elements in recent years. This research has led to significant improvements in quality and variety [1–3], driven by enhancing drilling efficiency and reducing costs.

This research has introduced new cutting teeth, including double-blade cutting teeth (TEC), chamfer edge strengthening teeth (CES), composite structure teeth (MC), and wedge teeth (SC). TEC teeth are equipped with two diamond cutting blades: a main blade and a secondary blade. Compared to traditional teeth, TEC teeth improve wear and impact resistance while reducing wear surface formation rates [4, 5]. The primary technical feature of Pre-chamfer edge strengthening teeth is the chamfering of the cutting edge, with key parameters including taper C and length. CES teeth chamfer the edge, eliminating stress concentration and reinforcing the blade’s edge. This is particularly advantageous in reducing the likelihood of early-stage cutting tooth cracking failures when the bit is in use [6]. MC teeth have a diamond layer, a PDC claw structure, and a natural diamond pregnancy insert. The claw structure enhances the connection between the diamond layer and the tungsten carbide matrix, increasing the contact area and binding force. This, in turn, aids in heat dissipation and effectively mitigates the impact of thermal stress on the PDC composite sheet [7, 8].

SC teeth are typically used alongside standard PDC cutters in formations with higher compressive strength, where achieving pure shear drilling is challenging, resulting in improved drilling performance. To address the issues of rapid temperature increase and severe wear of drill bit cutter teeth in hard formations, experts have developed StayCool multidimensional cutting teeth [9]. These cutting teeth are designed to enhance thermal transfer efficiency on the cutter tooth’s surface, directing the heat generated during cutting toward removing rock cuttings. This innovative design effectively reduces the cutting teeth temperature, lowering the risk of thermal crack failures due to overheating. Simultaneously, it enhances cutting efficiency and extends the operational lifespan of the cutter teeth.

The unique cutting mechanism of multidimensional teeth offers new possibilities in designing drill bits for challenging drilling conditions. However, compared to conventional flat PDC drill bits, aspects still require further clarification, especially regarding the cutting mechanism and design guidelines for multidimensional teeth. Achieving or even surpassing the rotational speed levels of traditional flat inset teeth through profile design is a central challenge when aiming to replace inset composite cutters. Therefore, this paper, using a linear cutting finite element model, establishes cutting models for two types of multidimensional teeth, Ridge and Benz, alongside conventional flat teeth. Investigating the rock-crushing mechanism of multidimensional cutting teeth and analyzing the impact of varying cutting parameters on rock-crushing effectiveness provides a solid theoretical foundation for the widespread adoption and application of multidimensional teeth in drilling technology in the oil drilling industry.

### Models and equations

Building upon the geometric fundamentals of PDC drill bits [10, 11], the rock plastic constitutive relationship [12], rock strength criteria [13–15], and the rock damage mechanism [16, 17], we formulate a PDC single-tooth rock-crushing model through finite element analysis. Subsequently, we delve into the rock-crushing mechanism based on this foundation.

In the actual scenario of PDC tooth crushing rock, the rock is a nonhomogeneous material. This is primarily due to the presence of micro-cracks in the rock and variations in distribution in different directions. With changes in rock stress, its specificity also fluctuates. Simultaneously, the plastic flow of rock involves the slip between crystals, distinct from the crystal deformation observed in metal materials, imparting anisotropy to the rock. Consequently, in the interaction between PDC cutting teeth and rocks:

1. PDC cutting teeth are treated as rigid bodies, without consideration for tooth deformation;
2. The PDC cutting teeth and rock are treated as continuous materials, and defects such as gaps and microcracks in the rock are disregarded;
3. Ground stress and drilling fluid pressure are neglected;
4. Cutting is treated as an adiabatic process, and the influence of temperature on rocks and diamonds is overlooked;
5. The cutting process is perceived as the uniform cutting of PDC teeth, with the neglect of drill bit vibration.

### Geometric model

**Fig 1:**
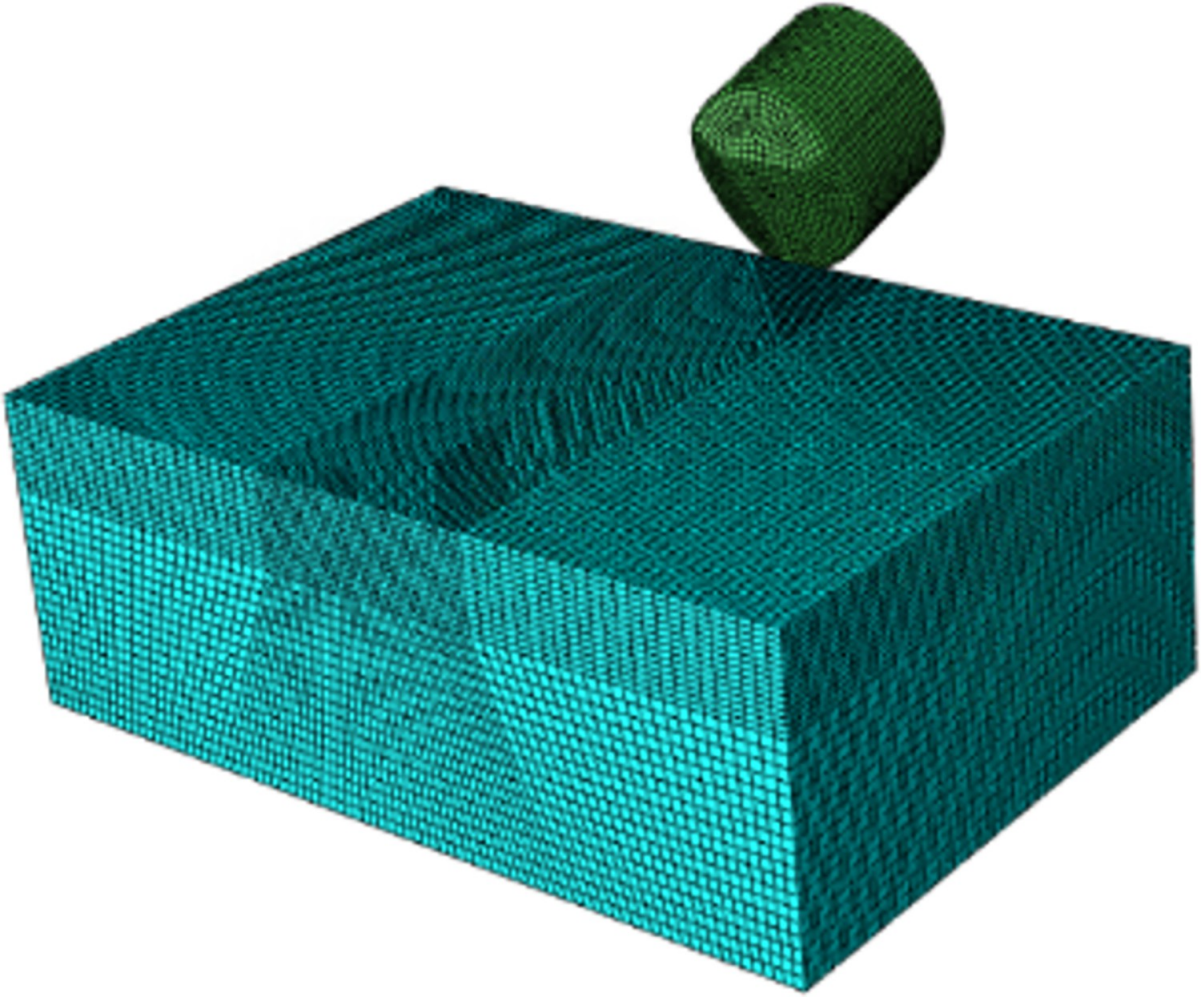
Line cutting model.

As shown in Fig 1, in modeling multidimensional rock, we have established single-tooth linear and core-turning models. Both multidimensional teeth and conventional flat teeth have a diameter of 16mm. Following the Saint-Venant principle, the rock model’s dimensions should be several times that of the tooth. Therefore, the linear cutting model measures 75mm in length, 25mm in width, and 25mm in height. All rock models mentioned above are partially segmented to facilitate subsequent local grid refinement.

### Grid division

The quality of grid division in the model plays a pivotal role in determining the calculation speed and accuracy of finite element analysis. A denser grid division, characterized by smaller mesh sizes, yields more precise calculation results but necessitates longer computation times. Conversely, coarser grids sacrifice some accuracy for shorter computational durations. Therefore, when partitioning the grid, we apply encryption to the region near the contact area with the cutting teeth, considering the limitations of existing hardware conditions and model characteristics. The size of this encrypted region is set to 0.4mm, while the grid spacing in other rock areas is 1mm. The chosen grid element is the eight-node linear reduction integral grid, denoted as C3D8R and an hourglass control is implemented to constrain grid transitions.

### Material attributes

The PDC cutting teeth and rock material parameters are shown in Table 1.

**Table 1:** PDC cutting teeth and rock material parameters.

Material	PDC	WuSheng	Beipei
Density/g·cm^-3^	15.4	2.54	2.46
Modulus of elasticity/GPa	890	11.52	31.75
Poisson ratio	0.077	0.062	0.118
Tensile strength/MPa	--	4.346	6.678
Shear strength/MPa	--	13.56	13.70
Compression strength/MPa	--	67.548	45.29
Internal friction angle/°	--	38.03	43.62

In this study, we adopt the extended Drucker-Prager model as the rock strength criterion and the shear damage model as the criterion for damage failure. The Drucker-Prager model considers the influence of intermediate principal stresses, resulting in increased material yield limits with increasing confining pressure. This characteristic eliminates singularity and enhances computational efficiency. Additionally, we introduce an extended Drucker-Prager parameter, k = 0.8, to approach the Mohr-Coulomb criterion. Notably, the rock shear dilation angle differs from the internal friction angle, adhering to the non-associated plastic flow law. This approach partially accounts for rock shear dilation while assuming limited hardening during the rock’s plastic stage. The criterion for shear failure is established based on the satisfaction of the Formulas (1) and (2). Subsequently, parameters related to equivalent effect variation, strain rate, and stress triaxiality at the onset of rock damage are set accordingly.


$$\varepsilon_{eq}^{**}=\frac{\varepsilon_{s}^{+}\sinh[f(\theta -\theta^{-})]+\varepsilon_{s}^{+}\sinh[f(\theta^{+}-\theta )]}{\sinh[f(\theta^{+}-\theta^{-})]}$$ (1)

$$\theta =\frac{1-k_{s}\eta }{\tau_{\max}}\sigma_{eq}$$ (2)

Where, *θ* is shear stress parameters, *k*_*s*_ is material parameters, *η* is stress triaxial degree, $\varepsilon_{eq}^{**}$ is strain of the first destruction, $\varepsilon_{s}^{+}$ and $\varepsilon_{s}^{-}$ are the equivalent pull/compression strain of the first damage, *θ*^+^ and *θ*^−^ are the *θ* parameters of equivalent tensile/compressive stress.

### Model constraints, contacts, and boundary condition

The reference point RP is set on the PDC tooth model, and the cutting tooth binds to the reference point through the rigid body constraint and changes into a discrete rigid body. The bottom and sides of the rock are set as the fixed constraints, and a speed load is applied to the reference point. The motion of the PDC rigid body is regarded as the motion of the reference point RP, and the cutting tooth cutting force is obtained by extracting the reaction force at the reference point.

Since the contact between the rock and the cutting teeth is a non-linear and relatively different deformation process, the contact surface of the rock should choose the node set of the whole rock model to avoid the situation that the cutting teeth only break the surface of the rock model and directly penetrate in the inside. The Contact Pair contact form of the rock grid and the cutting tooth grid is established in Interaction. The penalty function formula of elastic slip is adopted, the tangential friction factor is 0.4, and the direction of the contact surface adopts the Hard Contact formula.

Typical multidimensional teeth include the Ridge tooth and the Benz tooth, and their important performance indexes, such as rock breaking efficiency and force state, are jointly determined by the structural parameters of cutting teeth, working forward inclination Angle, axial rotation Angle, tooth rotation Angle, and formation properties. Choosing the appropriate cutting parameters for cutter teeth based on specific working conditions is critical in drill bit design and manufacturing. Based on the finite element numerical simulation, this section will study the change rules of rock breaking efficiency and force state of two typical multidimensional teeth under different cutting parameters. It provides some references for selecting multidimensional tooth bits and designing new multidimensional teeth in the Changqing area.

### Multidimensional tooth structure parameters

**Fig 2:**
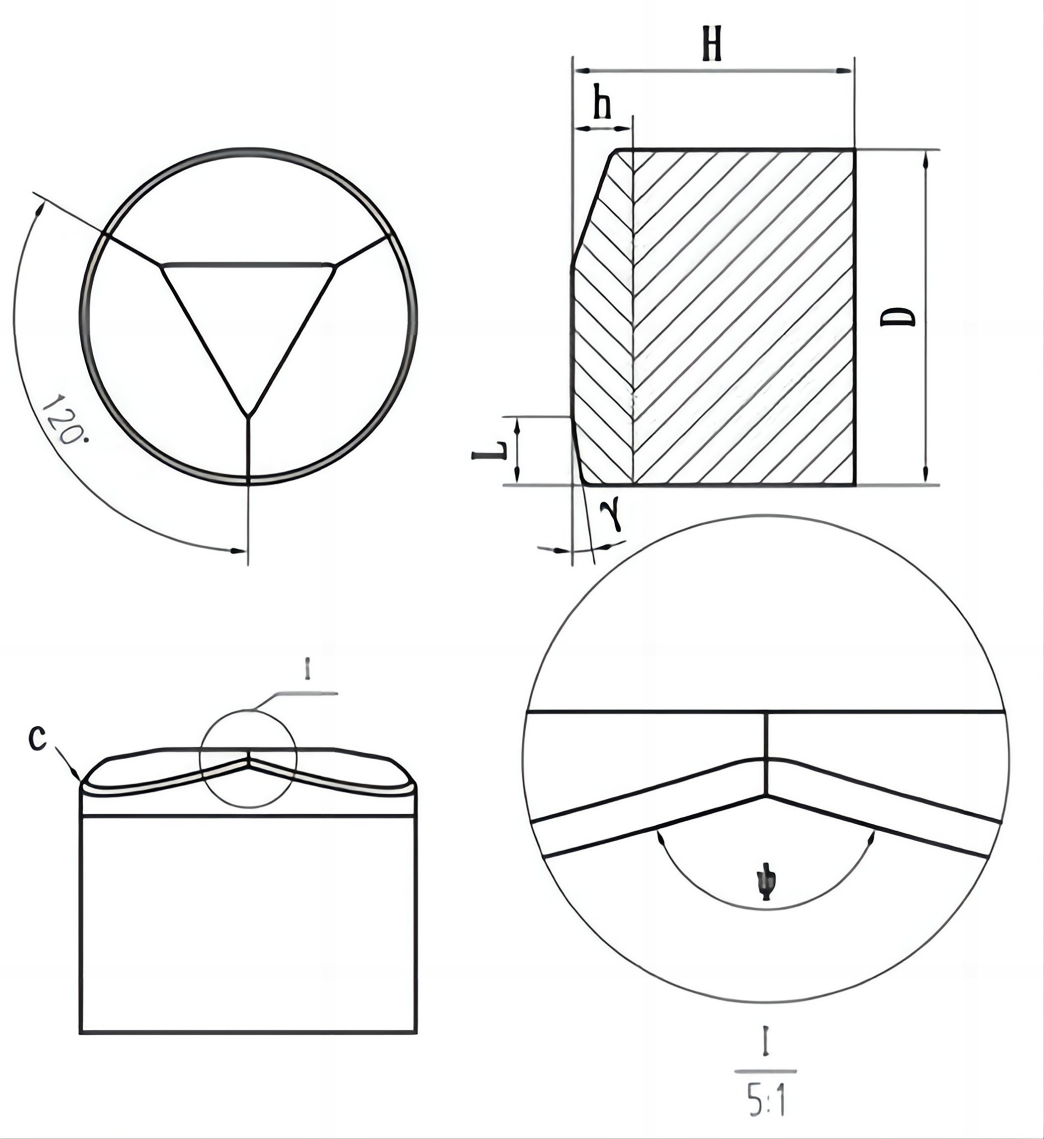
Benz tooth structure parameters.

**Fig 3:**
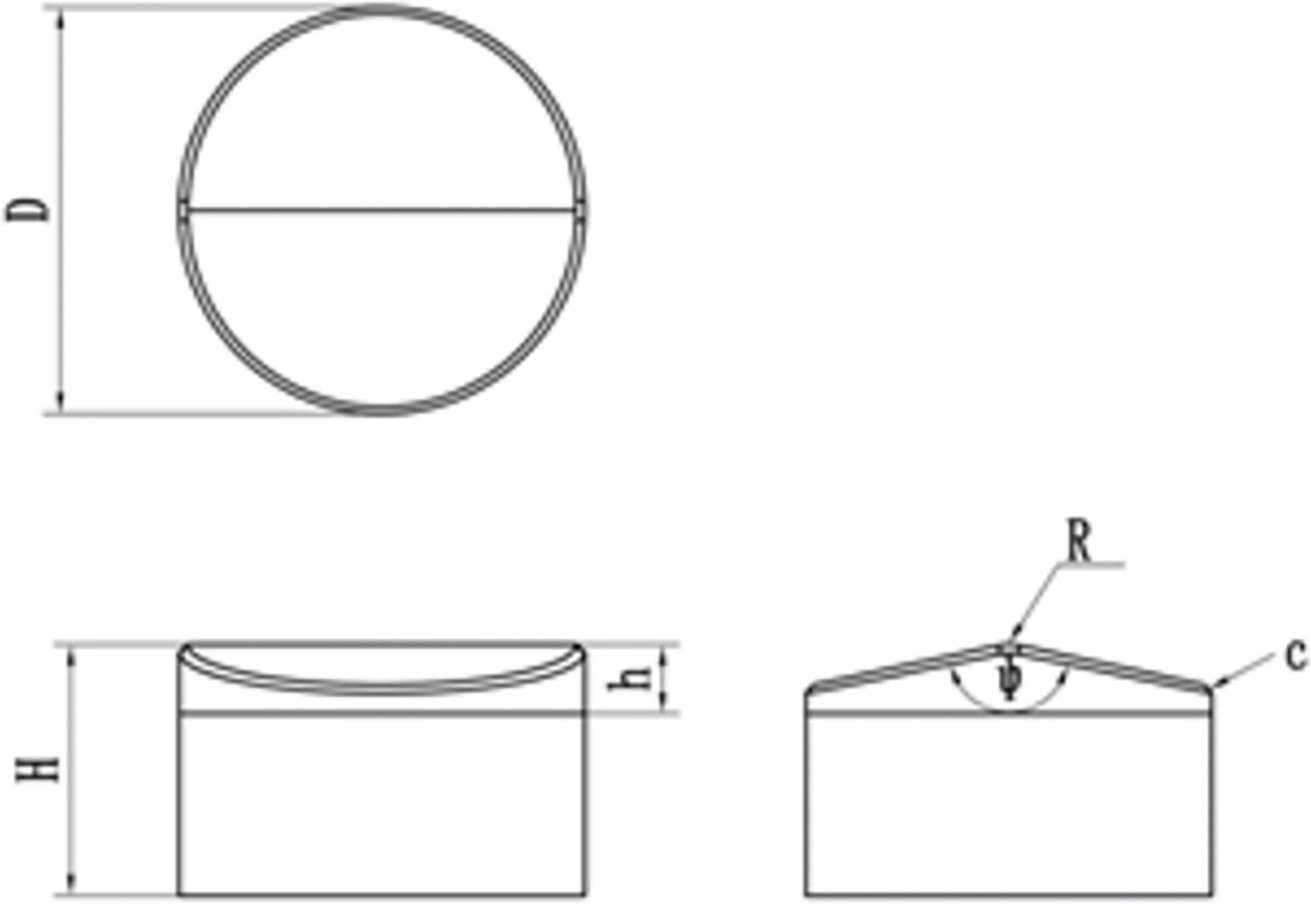
Ridge tooth structure parameters.

The work in this section focuses on the structure of two multidimensional teeth to find the structural parameters with the most significant impact on the multidimensional tooth breaking performance in Figs 2 and 3. The main structural parameters of the two multidimensional teeth are diameter D, height H, back angleψ, Ridge angleγ, Ridge slopeγ, Ridge length L, inverted angle R and inverted angle c.

The operating parameters of cutting teeth front rake, side turn angle, and cutting depth are consistent with the structural parameters. At the same time, ignoring the influence of surrounding pressure, temperature, and drilling fluid, the cutting tooth-breaking process under different structural parameters is simulated based on the linear cutting model established in ABAQUS. The obtained simulation results are used to judge whether the influence of each structural parameter on the tangential force and tangential force vibration is significant.

### Numerical simulation study of typical multidimensional tooth linear cutting

To reduce the amount of calculation, reduce the time cost. In the following numerical simulation, the geometric parameters of both multidimensional teeth are ψ = 156, back angle R = 2mm, inverted angle c = 0.4mm, Ridge length L = 3mm, and Ridge slope γ = 3.

Currently, the breaking efficiency is usually evaluated by the energy (Mechanical crushing ratio work MSE) consumed per unit volume of rock.


$$MES\approx \frac{E}{V}$$ (3)

Where: E is the energy consumed of broken rock, V is the volume of broken rock.

For a single cutting tooth, the calculation of rock crushing volume can be categorized into projection and actual volume [18–20]. The projected crushing volume results from multiplying the projected area of the cutting tooth by its displacement, whereas the actual crushing volume represents the physical volume of the removed rock. Due to its simplicity and divergence from the actual volume, the numerical simulation calculates the specific energy for rock crushing based on the projected volume. The specific energy associated with the projected crushing volume can be expressed as follows [21–23]:

$$MSE\approx \frac{E}{V}=\frac{f_{1}v_{1}+f_{2}v_{2}+f_{3}v_{3}}{Sv_{1}}\approx \frac{f_{1}}{S}$$ (4)

Where, *f*_1_, *f*_2_, *f*_3_ are tangential force, axial force and axial force, respectively, *v*_1_, *v*_2_, *v*_3_ are tangential, axial, and axial velocity, respectively, S is the projection area of the cutting teeth.

The projected area S of the conventional plane tooth is:

$$S=[r^{2}\cos^{-1}(\frac{r-d/\cos\theta }{r})-(r-\frac{f}{\cos\theta })r\sin(\cos^{-1}(\frac{r-d/\cos\theta }{r}))]\cos\theta$$ (5)

Where, r is the cutting the tooth radius, d is the eating depth of the cutting teeth, *θ* is the front rake.

## Results and discussion

### Numerical simulation of rock-crushing process with different multidimensional tooth structure parameters

#### Front rake

The size of the front rake in PDC cutting teeth significantly determines the cutting teeth’ rock-crushing efficacy, impact resistance, and wear resistance. Typically, a smaller front rake enhances the aggressiveness of the cutting teeth, resulting in better rock-crushing efficacy but making them more susceptible to fracturing and wear failure. Conversely, when the front rake is larger, cutting teeth exhibit enhanced resistance to impact and wear, but their aggressiveness decreases, leading to lower rock-crushing efficacy.

**Fig 4:**
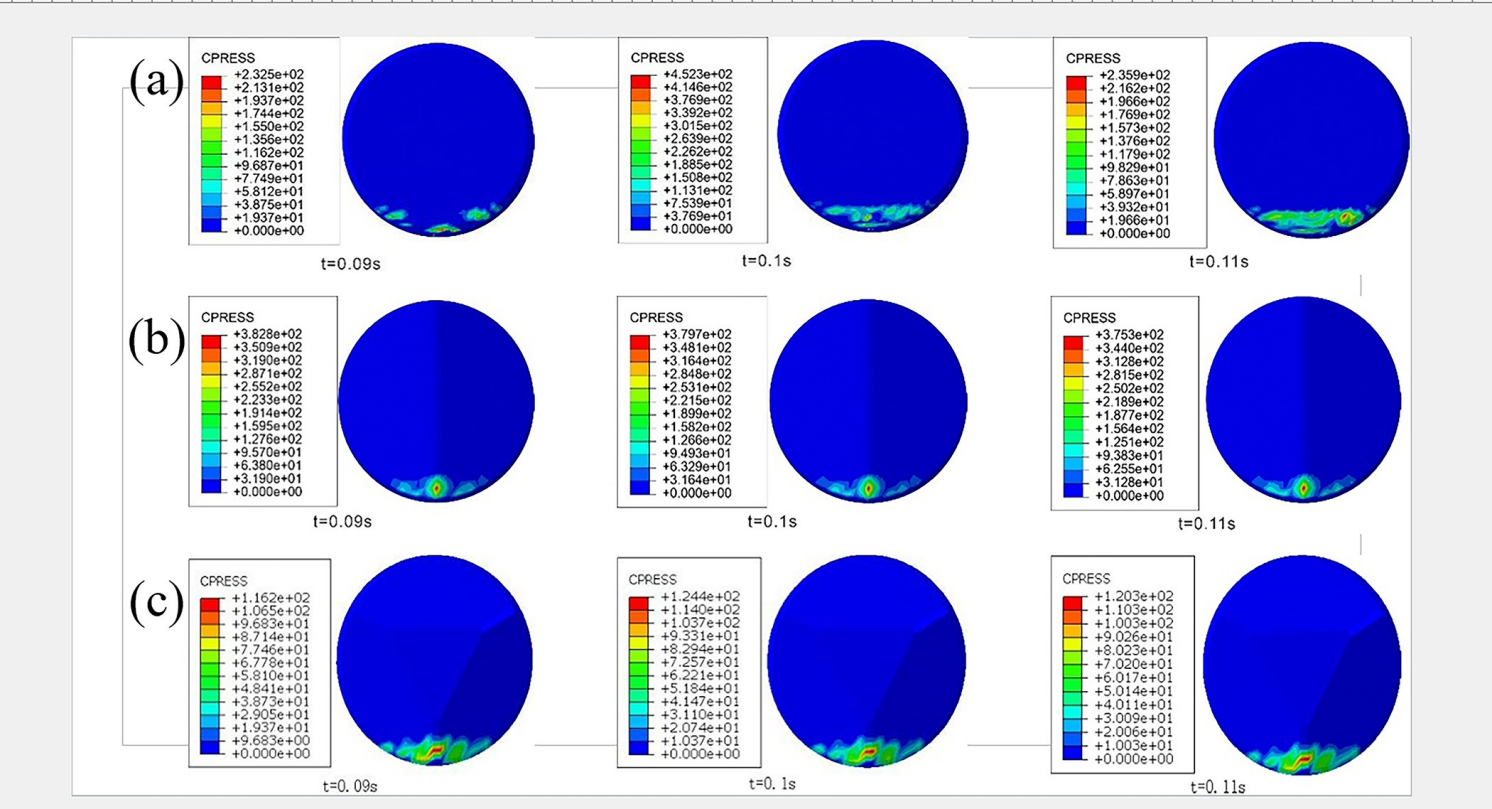
Cloud map of different front rake cutting teeth: (a)conventional planar teeth; (b)Ridge tooth; (c)Benz tooth.

In tooth placement design, selecting a front rake for different teeth and positions on drill bit blades also becomes particularly important. In the linear cutting model of Wusheng sandstone, the contact stress Cpress distribution on cutting teeth during the rock-crushing process at a front rake of 15° is illustrated in Fig 4. Cpress stress is defined in ABAQUS as occurring exclusively at the interaction interface between the rock and the cutting teeth, with zero stress values in other regions. It reflects the load distribution on the cutting teeth and the size of the contact area with the rock, also known as contact stress.

Fig 4 shows that conventional planar teeth exhibit significant variations in the contact stress region during the cutting process, with three distinctly different contact stress regions at different time intervals. The variations in the contact stress region are primarily attributed to the rock reaching its failure conditions during the cutting process, losing cohesion with the rock matrix, forming rock cuttings, and no longer making contact with the cutting teeth. Furthermore, during the rock-crushing process, conventional planar teeth exhibit a random pattern in rock fragmentation due to the planar shape of the diamond layer. This leads to an unstable contact during the cutting process, potentially resulting in significant cutting force and impact vibrations. Uneven contact also increases the likelihood of diamond layer detachment and rapid wear, among other failure scenarios.

**Fig 5:**
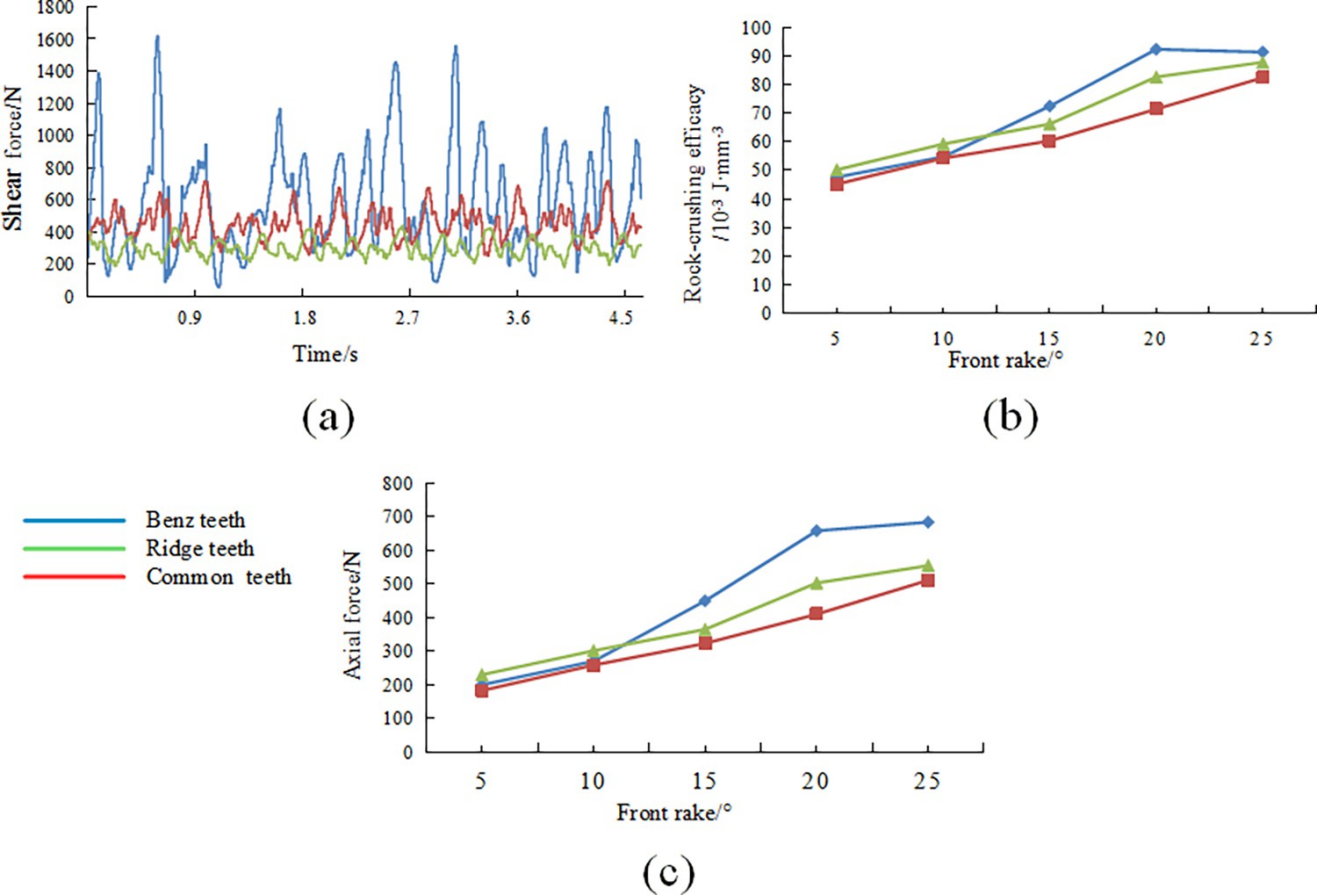
Change of cutting state during the rock breaking process (front rake15): (a) shear force variation curve of cutting teeth; (b) crushing specific function variation of cutting teeth; (c) variation of axial force of cutting teeth.

In order to precisely compare the variations in cutting states during the rock-crushing process for different cutting teeth, line charts depicting changes in cutting forces over time are extracted for three types of cutting teeth at a front rake angle of 15°. Fig 5 shows a significant oscillation in cutting forces over time, primarily due to the rock reaching its failure conditions, resulting in fragmentation and fracture. Some elements fail, absorbing plastic deformation energy, leading to a rapid decrease in cutting forces. However, as cutting needs to continue in the subsequent element, the cutting forces increase again. Fig 5A shows that the tangential force fluctuations of conventional planar teeth are significantly larger than those of the two non-planar cutting teeth. As described in the analysis of the contact stress contour map, conventional planar teeth exhibit pronounced tangential force fluctuations due to unstable contact during the rock-crushing process, resulting in significant vibrations during drilling and making them more susceptible to impact damage on the cutting teeth. On the contrary, due to their unique Ridge structure, multi-dimensional teeth experience smaller fluctuations in cutting forces during homogeneous sandstone fragmentation, which is beneficial for reducing drill bit impact and torsional vibrations.

At a cutting depth of 1.5mm, the variation in rock-crushing efficacy and axial forces with changing front rake is illustrated in Fig 5B and 5C. As depicted in Fig 5, at lower front rake angles, there is minimal difference in the required rock-crushing efficacy and axial forces among the three types of cutting teeth. However, when the front rake angle exceeds 10°, conventional planar teeth exhibit significantly higher requirements for rock-crushing efficacy and axial forces than the two multi-dimensional teeth. The Ridge’s tooth demands the least rock-crushing efficacy and axial forces. In existing drilling operations for rock-crushing, the impact and wear on the cutting teeth at the nose and shoulder of the drill bit are common causes of bit failure. Therefore, during drill bit tooth layout design, a relatively larger front rake angle is often chosen for this region. Consequently, it can be inferred that compared to conventional planar teeth, the two multi-dimensional tooth designs require lower drilling pressures for breaking rock at the drill bit’s nose and shoulder, making it easier to penetrate the geological formations. Additionally, the lower rock-crushing efficacy suggests lower torque requirements during actual drilling, which is advantageous for reducing the stick-slip vibrations of the drill bit and enhancing the application of PDC drill bits in the build-up section.

#### Side turn angle

The purpose of designing the side turn angle for PDC drill bit cutting teeth is to facilitate the removal of cuttings, but it also affects the stability of the drilling process. Inappropriate side turn angles may lead to axial vibrations of the drill bit, tooth breakage, tooth detachment, and other unfavorable conditions. However, due to the multidimensional nature of the diamond surface, multidimensional teeth experience a more complex stress state during rock cutting.

**Fig 6:**
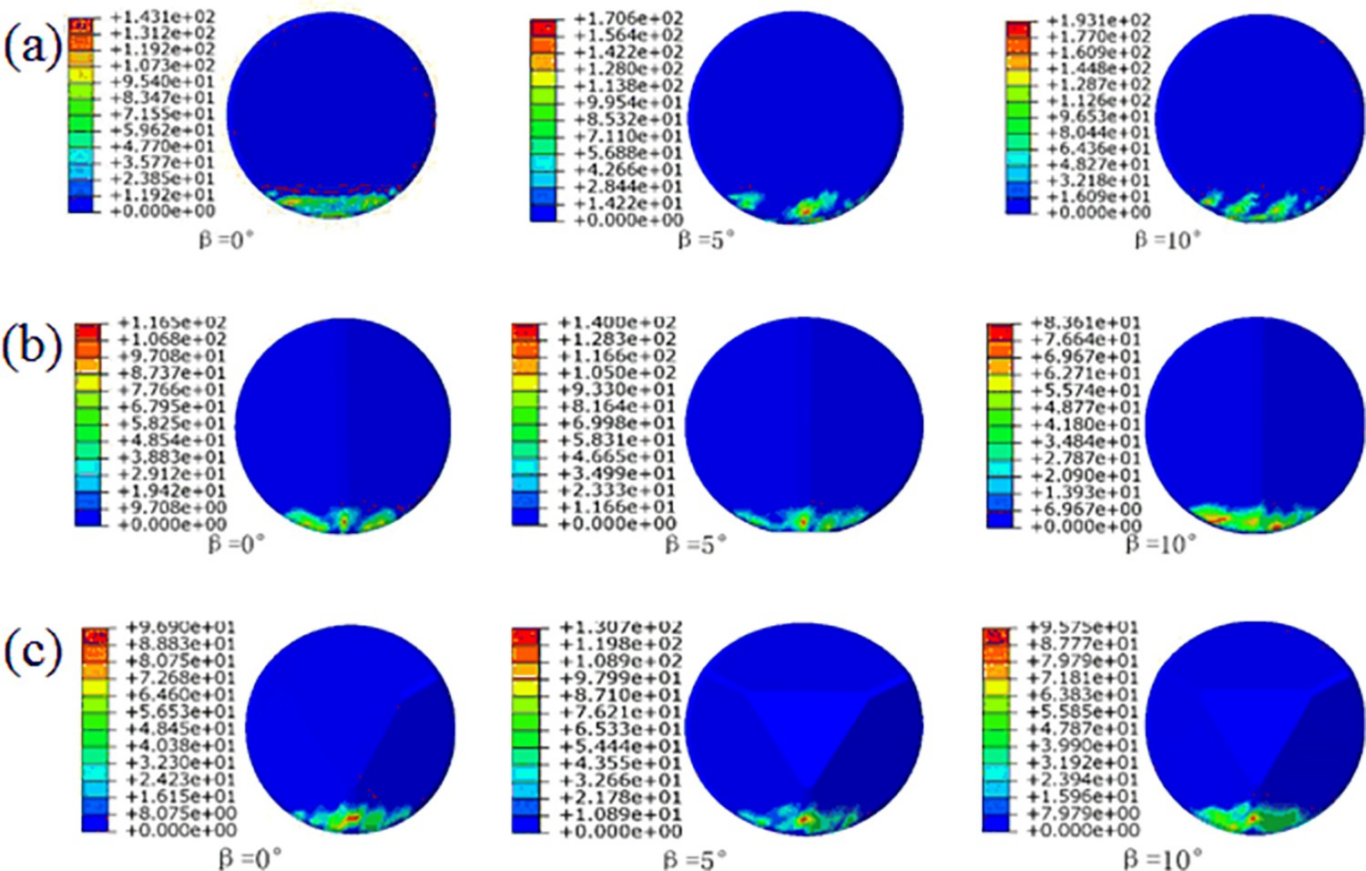
Cloud map of different side turn angle cutting teeth: (a)conventional planar teeth; (b)Ridge tooth; (c)Benz tooth.

Fig 6 illustrates the contact stress (cpress) distribution throughout the entire linear cutting process for three types of cutting teeth under different side turn angles (β) when the front rake is set at 15°. When a significant side turn angle (β) is present, conventional planar teeth exhibit noticeable variations in the contact area with the rock. The uniformity of contact stress distribution is poor, and significant cutting force vibrations may occur during rock fragmentation.

In contrast, for Fig 6B and 6C, the two types of multidimensional teeth show no significant reduction in the contact area, even in the presence of a noticeable side turn angle. This indicates that multidimensional teeth maintain good contact with the rock, and the cutting force vibrations are relatively small, even with a significant side turn angle. However, it is also evident that the contact stress area tends to shift toward one side of the Ridge, suggesting potential differences in stress distribution on both sides of the multidimensional Ridge teeth.

**Fig 7:**
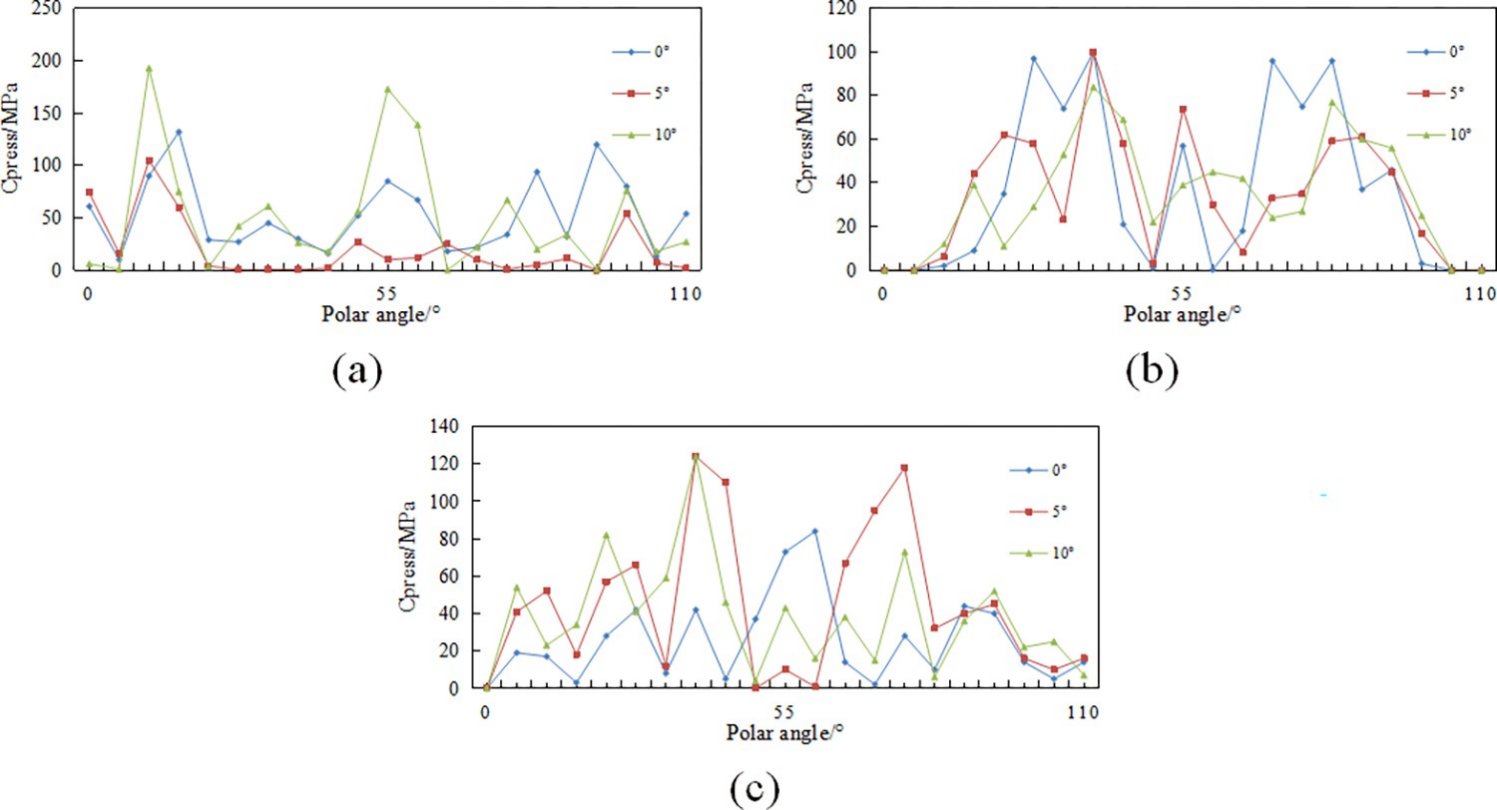
Contact stress variation rule of tooth blade: (a)conventional planar teeth; (b)Ridge tooth; (c)Benz tooth.

To better understand the changing stress state during the rock-cutting process of the cutting teeth, we establish a polar coordinate system based on the average contact stress distribution contour map. Taking the Benz tooth as an example, the polar coordinates of the cutting teeth were defined with the cutting tooth axis as the axis within the normal plane, setting it as the coordinate origin O. The ray at the polar angle of 55° coincides with the ridge line of the Benz tooth. The extracted node range covers 0° to 110°, as shown in Fig 7.

From the contact stress contour maps in Fig 6, the variations in the distribution pattern of contact stress along the cutting tooth edge nodes in polar coordinates were obtained, as shown in Fig 7. In Fig 7A, it can be observed that conventional planar teeth exhibit higher contact stresses primarily at the bottom and sides of the tooth edge, aligning with conclusions drawn from the study of rock-cutting mechanisms involving conventional planar teeth at the tooth edge. Additionally, it is evident that with an increase in the side turn angle, conventional planar teeth experience an increase in contact stress at polar angles of 5–25° and 40–60°. This indicates that the side turn angle shifts the load distribution of the tooth edge toward one side, resulting in asymmetry. The distribution patterns of the two types of multidimensional teeth in Fig 7B and 7C reveal that contact stresses concentrate around the ridges of multidimensional teeth and in the vicinity of both sides of the ridge. This aligns with the conclusions drawn from the study of rock-cutting mechanisms, where rock stresses concentrate at central positions.

Furthermore, with an increase in side turn angle, there is a noticeable increase in contact stress values at polar angles of 20–35° and 65–80° on both sides of the ridge of the Benz tooth. This indicates that in the presence of a side turn angle, the region where the Benz tooth edge generates stress concentration on the rock shifts from along the ridge to the vicinity of both sides of the ridge. However, both types of multidimensional teeth maintain a symmetrical distribution of contact stress even after an increase in side turn angle, specifically at polar angles of 55° on both sides of the ridge. This suggests that compared to conventional teeth placed at the nose or shoulder of the drill bit, the arrangement of multidimensional teeth with side turn angles reduces the likelihood of tooth breakage, detachment of diamond layers, or tooth loss. Additionally, it may generate smaller axial forces, reducing the occurrence of bit bouncing and axial vibrations.

**Fig 8:**
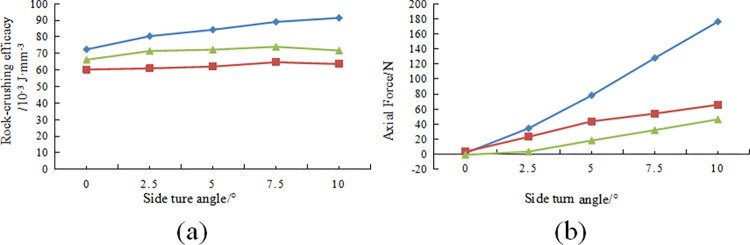
Different side turn angle cutting state changes in the cutting tooth breaking process: (a) Change variation of different side turn angle cutting teeth; (b) Change law of the axial force of different side turn angle cutting teeth.

**Fig 9:**
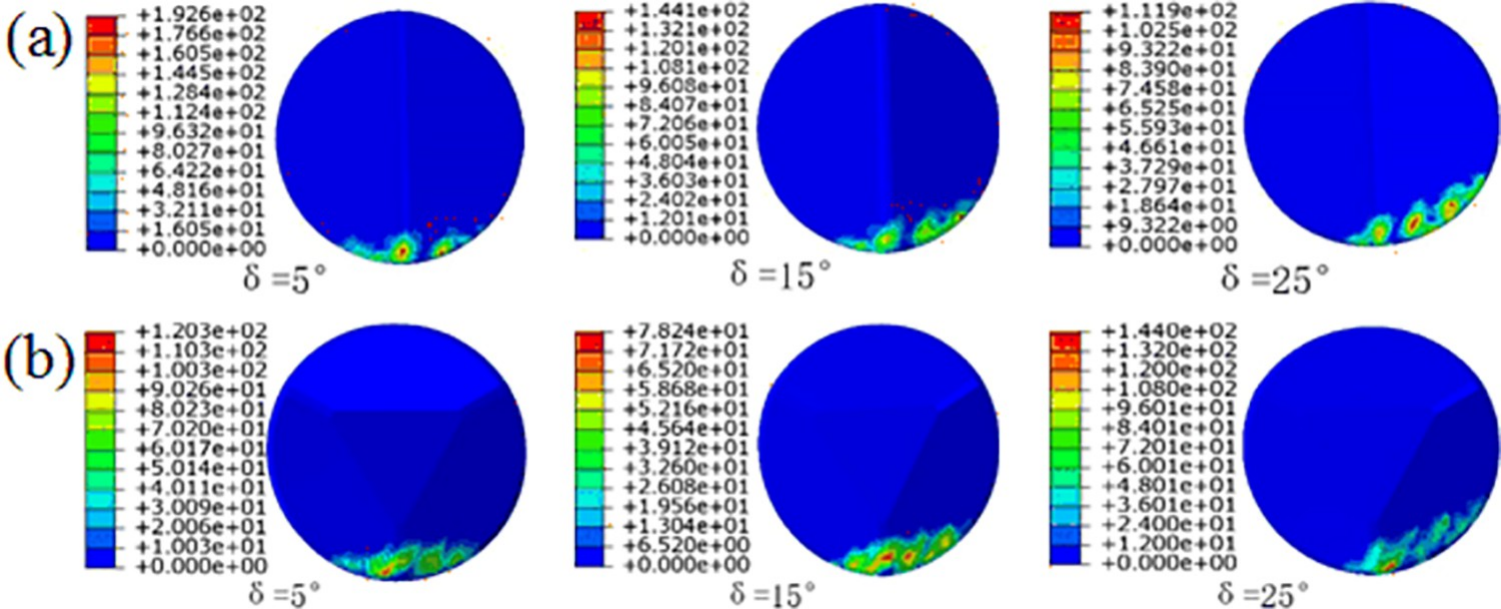
Contact stresses of different tooth spin angle cutting teeth: (a)Ridge tooth; (b)Benz tooth.

During the linear cutting process, the variations in rock-crushing efficacy of the cutting teeth under different side turn angles with a front rake of 15 degrees are shown in Fig 8A. The variations in axial forces of the cutting teeth under different side turn angles with a front rake of 15 degrees are depicted in Fig 9B.

Fig 8A shows that conventional planar teeth require higher rock-crushing efficacy than multidimensional teeth under different side turn angles, with Ridge teeth requiring the least specific energy for rock breaking. As shown in Fig 8B, axial forces significantly increase with an increase in side turn angle, and conventional planar teeth exhibit higher axial forces than the two types of non-planar cutting teeth. It can be inferred that the planar structure of the diamond layer of conventional planar teeth hinders the rock-cutting efficiency when side turn angles are present, whereas side turn angles have a relatively minor impact on the rock-cutting efficiency of the two types of multidimensional teeth. At the same time, side turn angles have a notable effect on the axial forces of the cutting teeth, and conventional planar teeth experience a faster increase in axial forces compared to multidimensional teeth. Considering the contact stress data results, it can be anticipated that larger side turn angles are more likely to result in situations where cutting teeth break or detach. For multidimensional teeth, uneven loading on both sides of the ridge can also lead to detachment of the diamond layer, and non-uniform wear on both sides can quickly reduce the rock-cutting efficiency of the cutting teeth.

#### Tooth spin angle

In addition to conventional tooth parameters such as forward inclination and axial turn, the Ridge tooth and Benz tooth also have unique ridge structure requirements. This requirement necessitates that after drill bit manufacturing, the multi-dimensional tooth ridges should be perpendicular to the rock surface or, in other words, aligned with the normal vector of the cutting tooth envelope surface. However, achieving precise welding requirements due to manufacturing constraints increases the drill bit’s manufacturing cost and is also challenging to accomplish. Therefore, due to the inevitable welding errors, there are widespread discrepancies in the orientation of the multi-dimensional teeth arranged in PDC drill bits. This angle is defined as the tooth spin angle and is denoted as. The impact of the tooth spin angle on the rock-crushing efficacy, impact resistance, wear resistance, and other performance aspects of multi-dimensional teeth remains unclear. Investigating the influence of this angle on the rock-crushing process with multi-dimensional teeth is urgently needed.

In the linear cutting model, the primary focus is on the cutting teeth located at the shoulder and nose of the drill bit, where the cutting teeth have a larger radius of rotation. These cutting teeth at these positions experience higher linear velocities and typically undergo the most severe wear on the drill bit, which is why they often have the highest tooth density on the drill bit. Consequently, the cutting teeth at these locations usually have smaller arc lengths of contact with the rock and smaller contact areas on the working face. For multi-dimensional teeth, the impact of welding errors resulting in different δ angles is more pronounced. When the front rake is 15°, under different tooth spin angle δ conditions, the average contact stress and compress in the linear cutting of Ridge’s tooth and Benz’s tooth are shown in Fig 9.

Fig 9 shows that as the tooth spin angle δ increases, there are significant changes in the contact area between the cutting teeth and the rock. When the tooth spin angle δ = 5°, the contact stress area of the multi-dimensional teeth does not show significant differences compared to the case with no tooth spin angle. A noticeable area of high stress can still be observed at the ridge structure, indicating the generation of point loads on the rock through the ridge structure. There is also some uneven stress distribution on both sides of the Ridge. However, at tooth spin angles δ = 15° and 25°, there are significant differences in the contact stress areas of the two types of multi-dimensional teeth compared to the conditions with tooth spin angles of 0°or 5°. Severe uneven stress distribution is observed on both sides of the Ridge, and the contact stress areas are discontinuous, with stress concentration areas shifting to one side. It can be inferred that multi-dimensional teeth exhibit a rock-crushing effect similar to the ideal condition with a tooth spin angle of 0° under smaller tooth spin angles. This effect is achieved through point loads and shearing action generated by the ridge structure. However, when welding errors result in a significant tooth spin angle, the concentration of contact stress areas does not only occur at the Ridge. The shift in stress concentration areas may lead to the initial wear of cutting teeth occurring at the edges of the diamond layer on the inclined surfaces or the working face rather than at the ridge structure of the diamond layer. The diamond layer on the ridge structure of multi-dimensional teeth is typically thicker, usually in the range of 2.5-3mm, whereas the diamond layer on the edges of the inclined surfaces is thinner, typically in the range of 1-2mm. Excessive shifting of contact stress may result in rapid wear to the hard alloy matrix at the edges of the inclined surfaces, consequently reducing rock-crushing efficacy and premature failure of cutting teeth.

**Fig 10:**
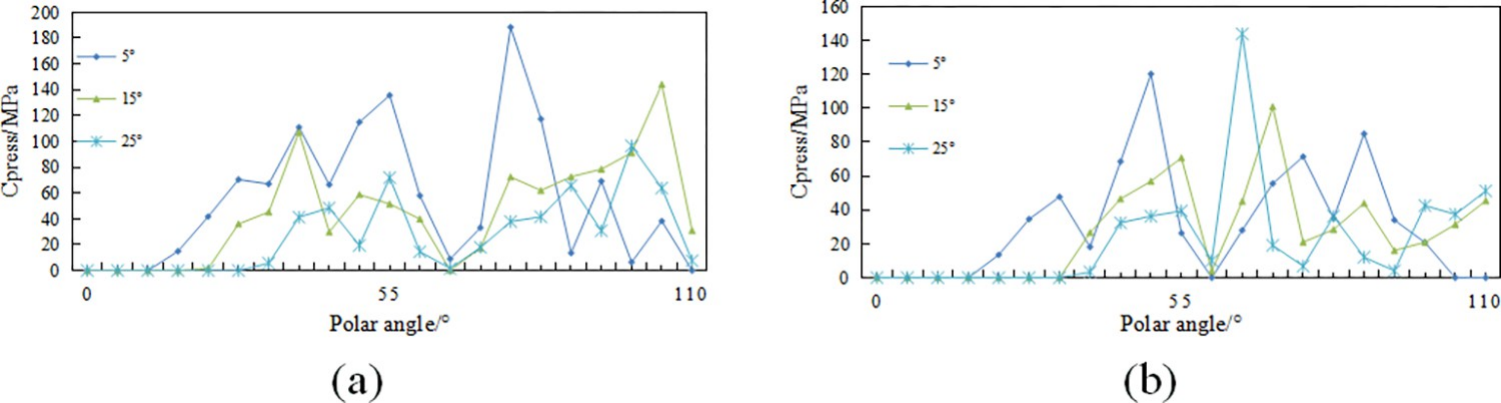
Change pattern of contact stress of different tooth spin angle tooth blades: (a)Ridge tooth; (b)Benz tooth.

To gain a more precise understanding of the distribution of contact stress on the cutting teeth, the contact stress distribution cloud chart at the cutting edge of multi-dimensional teeth is determined based on the contact stress contour map, as shown in Fig 10.

**Fig 11:**
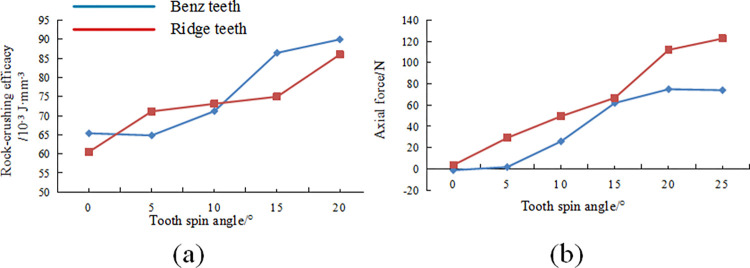
Changes of different tooth spin angle cutting state for cutting tooth breaking process: (a)Change law of the rock-crushing efficacy of different tooth spin angle cutting teeth; (b)Change law of the axial force of different tooth spin angle cutting teeth.

From Fig 11, it can be observed that the distribution of contact stress on both sides of the polar angle of 55°, namely on both sides of the ridge, is significantly imbalanced and uneven. At a tooth spin angle of 5°, the contact stress values and their extent on the side with cutting edge polar angles less than 55° are greater than those at tooth spin angles of 15° and 25°, and the contact stress is lowest in the vicinity of polar angles 60° to 70°. With an increase in tooth spin angle, the starting polar angle at which contact stress values appear also increases, indicating a shift and alteration in the segment of the cutting edge in contact with the rock.

At a front rake of 15° and a cutting depth of 1.5mm, the variation pattern of rock-crushing efficacy and axial forces for different tooth spin angles δ are shown in Fig 11A and 11B, respectively. Fig 11A shows that the presence of tooth spin angle significantly affects the variation in rock-crushing efficacy, with an increase in tooth spin angle leading to an increase in the rock-crushing efficacy for multi-dimensional teeth. For Ridge tooth, the rock-crushing efficacy steadily increases within the range of tooth spin angle 0–10°, with a relatively small increase, while within the range of tooth spin angle 10–20°, the required rock-crushing efficacy for breaking rocks increases significantly. However, for Benz’s tooth, there is an increase in rock-crushing efficacy once the tooth spin angle is introduced.

The axial force variation pattern shows that the axial force increases significantly with tooth spin angle, and Benz’s tooth exhibits lower axial forces than Ridge’s tooth at the same tooth spin angle. These patterns indicate that multi-dimensional teeth’ unique tooth spin angle has a noticeable hindering effect on their rock-crushing efficacy. Larger tooth spin angles reduce the rock-crushing efficacy of cutting teeth and increase drilling costs. Furthermore, this leads to a significant increase in axial forces on the cutting teeth, which can easily lead to rapid wear of multi-dimensional teeth at the shoulder or nose, axial vibration of the drill bit, and ring-cutting issues. Therefore, in manufacturing drill bits, it is advisable to control the welding errors of multi-dimensional teeth within a smaller range of 0–10° and avoid the occurrence of larger tooth spin angles.

### Test and verification of rock-crushing effect

#### Experimental scheme

**Fig 12:**
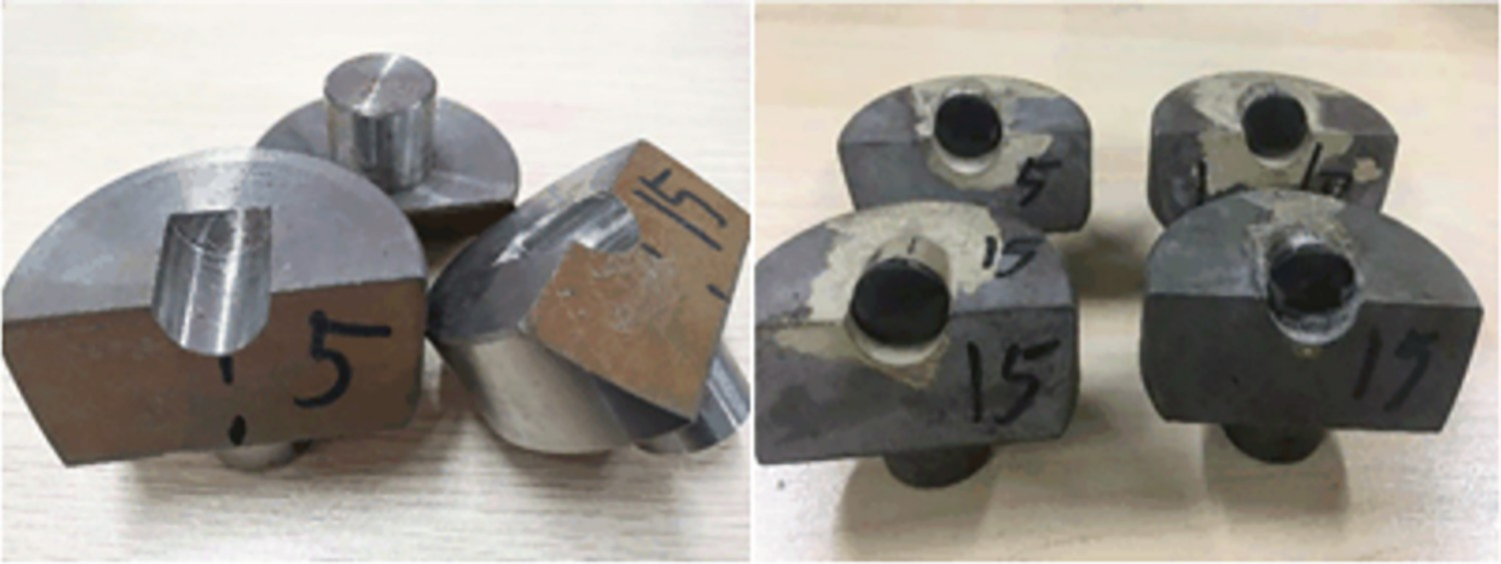
Cutting teeth and toothholder.

**Fig 13:**
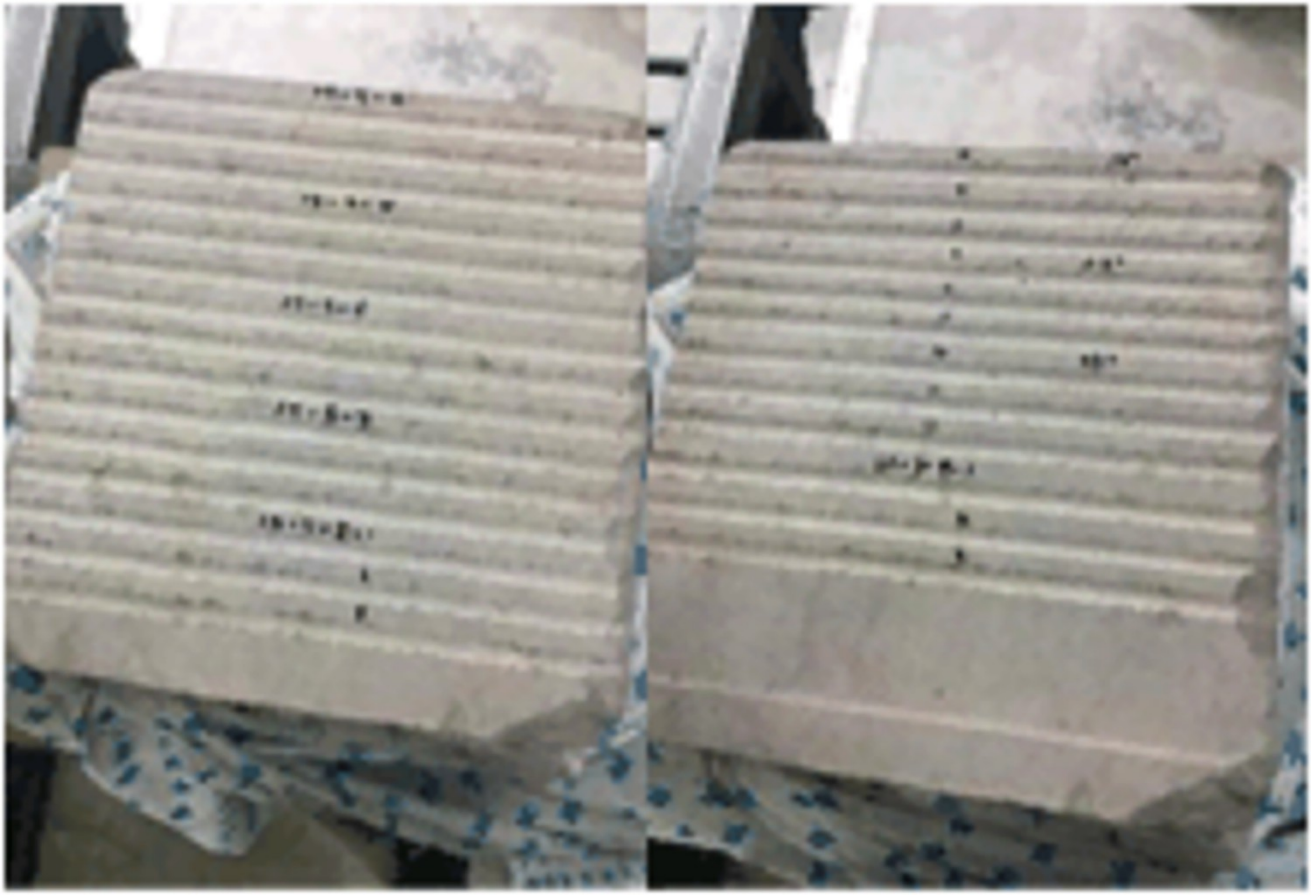
Test rock.

Conduct scraping experiments on Benz’s teeth with parameters such as front rake, side turn angle, tooth spin angle, and cutting depth. To reduce experimental errors and prevent incorrect results, perform three scrapes for each specific cutting parameter, the cutting teeth and toothholder are shown in Fig 12 and the test rock is shown in Fig 13.

1. Design and manufacture cutting tooth holders and fix the cutting teeth in the slots of the tooth holders through welding.
2. Obtain Benz teeth with different front rake angles (5°, 10°, 15°, 20°, 25°) by varying the angles of the tooth slots in the tooth holders. Use Benz teeth with different front rake angles to scrape Wusheng sandstone at cutting depths of 1.5mm, 2mm, and 2.5 mm and collect rock cuttings.
3. Vary the installation angle between the tooth holder and the cutter handle to obtain Benz teeth with front rake 15° and side turn angles of 0°, 2.5°, 5°, 7.5°, and 10°. Scrape Wusheng sandstone at a cutting depth of 1.5mm and collect rock cuttings.
4. During the welding process, deflect the Benz teeth at specific angles to obtain Benz teeth with a front rake of 15° and tooth spin angles of 0°, 5°, 10°, and 15°. Scrape Wusheng sandstone at a cutting depth of 1.5mm and collect rock cuttings.
5. Obtain cutting tooth cutting force data during the scraping experiments through a data collection system. Weigh the collected rock cuttings and calculate the volume of the cuttings using sandstone density. Calculate the specific mechanical energy of rock breaking for each scraping experiment using the formula from the rock-crushing efficacy evaluation criteria.

### Analysis of the test validation results

**Fig 14:**
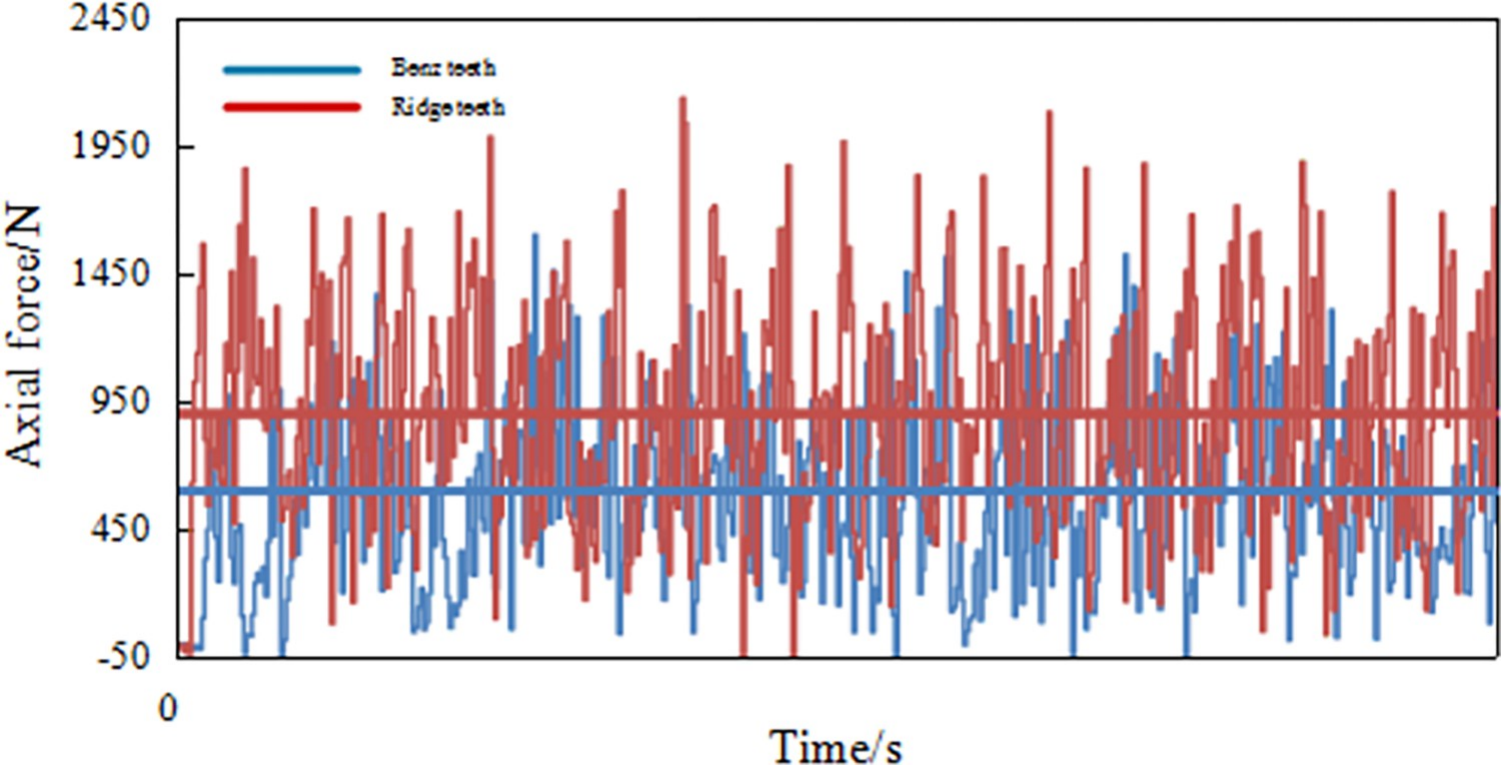
Changes of tangential force between Benz tooth and conventional teeth.

Through a data collection system, the changes in tangential forces for conventional planar teeth and Benz teeth at front rake 15° and cutting depth 1.5mm are shown in Fig 14. The data represents the average tangential force from a single scrape within three scraping trials, and the dashed line in Fig 14 represents the mean tangential force. From Fig 14, it can be observed that under the same cutting parameters, the average tangential force of conventional planar teeth is greater than that of Benz teeth.

By calculating the standard deviation of tangential force variations throughout the cutting process, it is found that the tangential force standard deviation for Benz teeth is 349.09N, while for conventional planar teeth, it is 439.53N. Under the same conditions, Benz teeth require lower tangential forces and experience less tangential force vibration when cutting and breaking sandstone compared to conventional planar teeth, indicating that Benz teeth experience less vibration impact when breaking rocks.

The obtained rock-crushing efficacy data from the experiments is compared with numerical simulations, and the error rate between the two is calculated. The error rate can indicate the degree of deviation between the actual values and simulated values in the results, and is calculated using the following formula:

$$E\mathrm{rror}=\frac{test-simulation}{simulation}$$ (6)

**Fig 15:**
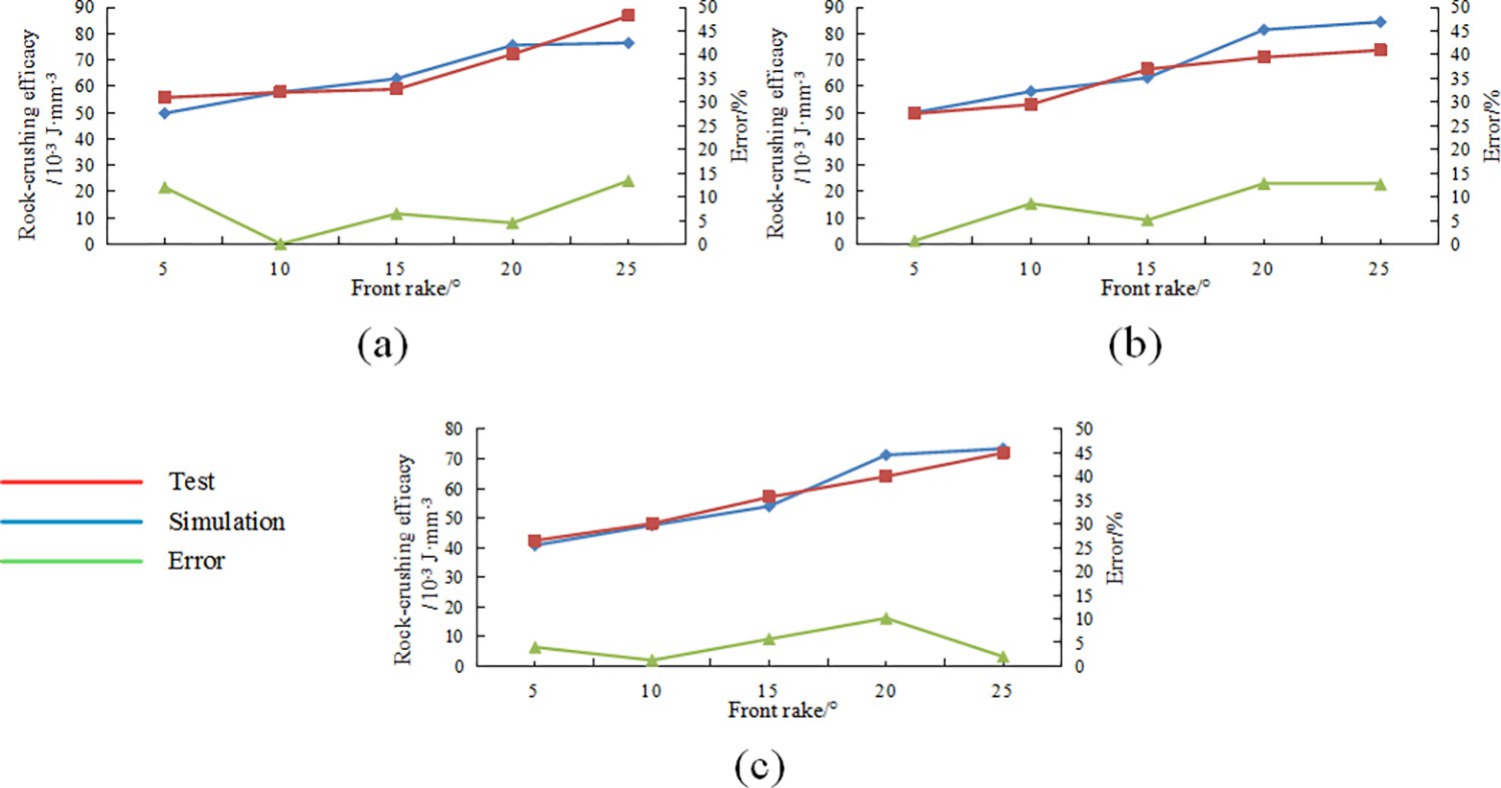
Comparing different front rake tests and simulation results: (a)cutting depth1.5mm; (b)cutting depth2mm; (c)cutting depth2.5mm.

The rock-crushing efficacy variation pattern of Benz teeth under different front rake angles and cutting depths obtained from scraping experiments is shown in Fig 15.

From the Fig, it can be observed that the rock-crushing efficacy increases with the increase in front rake in actual scraping experiments, consistent with the pattern in numerical simulations, and the error rate compared to numerical simulation results is within 15%.

**Fig 16:**
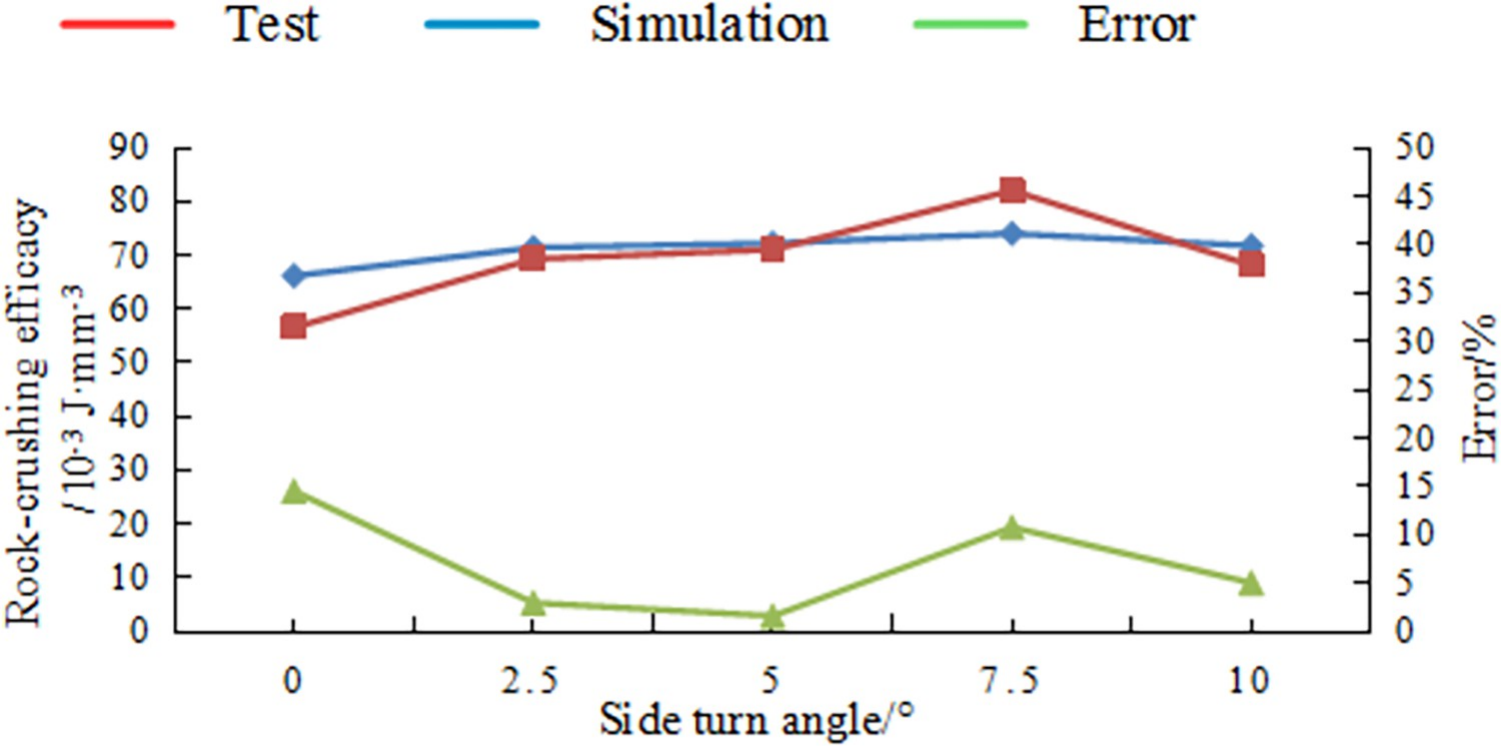
Comparison tests and simulation results different side turn angle.

**Fig 17:**
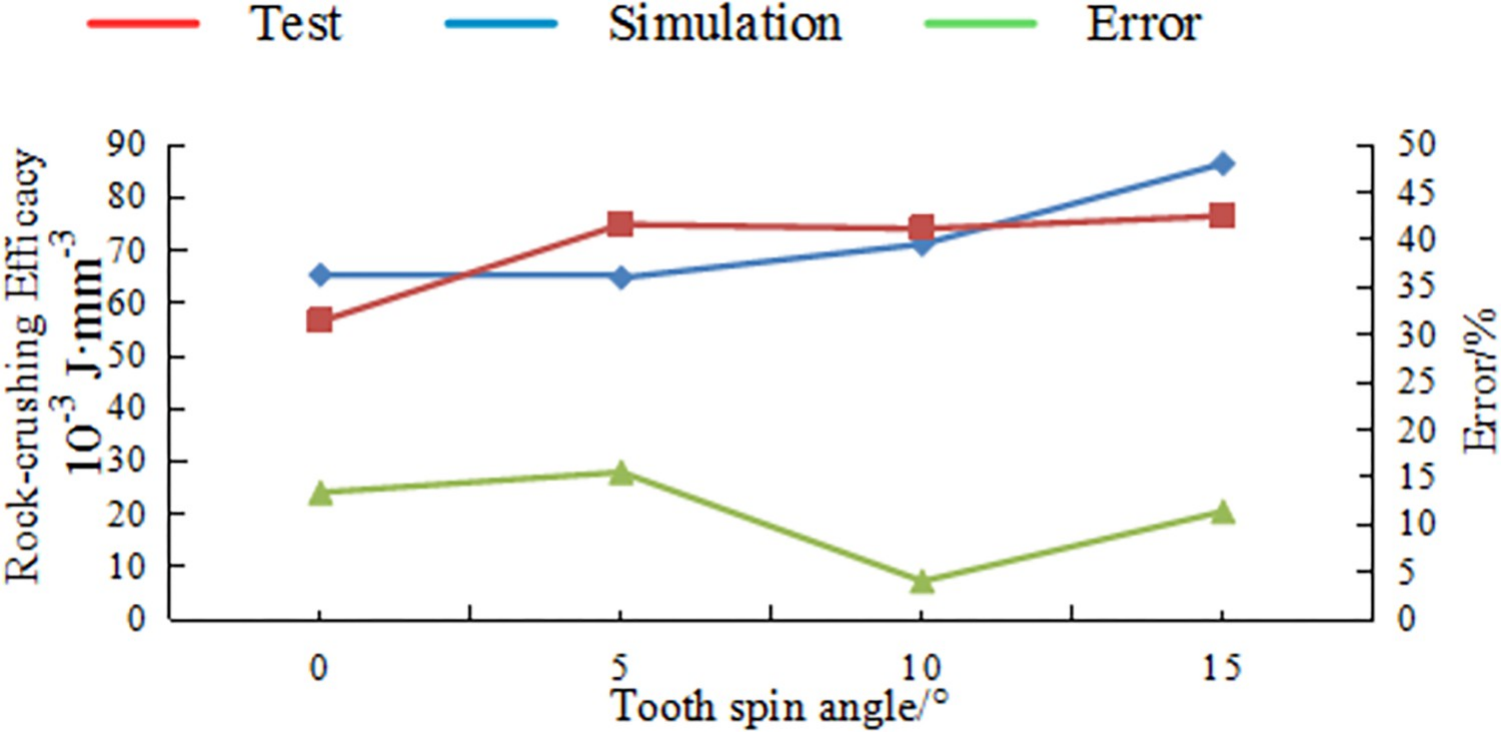
Comparison tests and simulation results different side turn angle.

Fig 16 illustrates the variation pattern of rock-crushing efficacy for Benz teeth with respect to side turn angle, while Fig 17 shows the variation in rock-crushing efficacy for Benz teeth with respect to tooth spin angle.

In the Figs, it is still noticeable that the experimental values closely match the numerical simulation values, with an error rate within 15%.

Therefore, it can be concluded that the rock-crushing efficacy obtained from numerical simulations closely aligns with experimental values, thus partially validating the reliability of the numerical simulation results.

**Fig 18:**
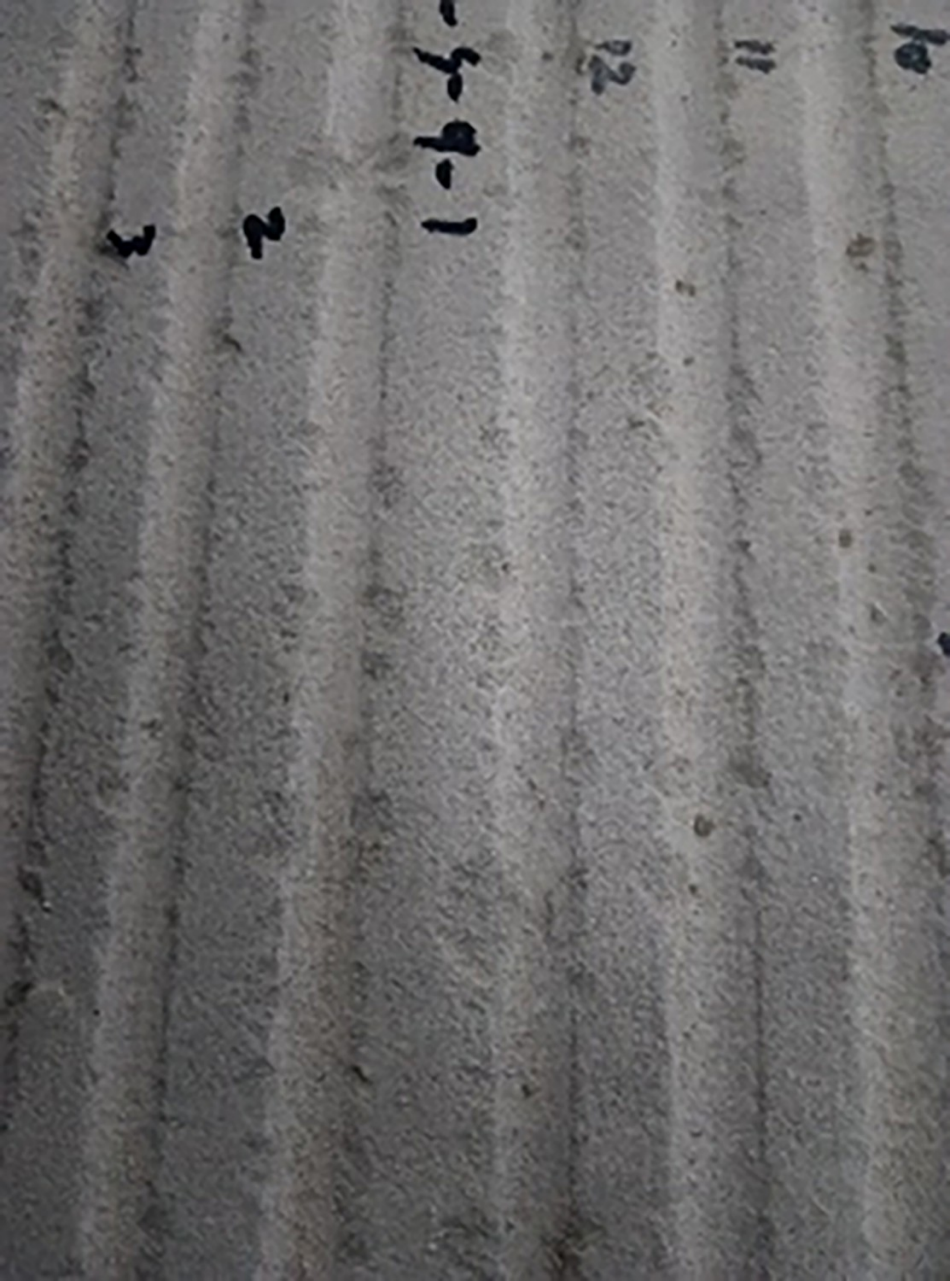
Rock side collapse.

During the test, it is found that the rock contacted by the cutting tooth edge collapsed somewhat (Fig 18). That is, the theoretical value of the rock volume of the cutting tooth is larger than the projected area, which reduces the rock-crushing efficacy and leads to a certain difference between the numerical simulation results and the actual value.

## Conclusion

This article establishes a linear cutting finite element model to investigate the cutting mechanisms of conventional planar teeth, ridge teeth, and Benz’s teeth. The main conclusions are as follows:

1. During the rock-crushing process with multi-dimensional teeth, the contact with the rock is stable, and cutting force vibrations are minimal. Benz teeth are more effective at penetrating the rock and creating fractures.
2. Multi-dimensional teeth have higher rock-crushing efficacy than conventional planar teeth. When positioned at the shoulder and nose of the drill bit, they can reduce drill bit torque to some extent while improving rock-crushing efficacy. Lower torque also reduces drill bit stick-slip vibrations and enhances the drill bit’s directional drilling capability.
3. Compared to conventional planar teeth, Benz teeth exhibit lower cutting forces and reduced cutting vibrations when breaking rocks. This reduces drill bit torque and minimizes stick-slip vibrations, enhancing drill bit impact resistance and wear resistance.
4. The tooth spin angle reduces the rock-crushing efficacy of Benz’s teeth and intensifies cutting vibrations. It is advisable to avoid excessive tooth spin angles during drill bit manufacturing.
5. With increased cutting depth, tooth vibrations significantly increase, leading to larger-sized rock cuttings. This indicates that significant rock cutting occurs only at specific depths.

## References

[1] Zhang Z, Zhao D, Zhao Y, et al. Simulation and experimental study on temperature and stress field of full-sized PDC bits in rock breaking process. Journal of Petroleum Science and Engineering, 2020, 186: 106679.

[2] Tian H, Ren H, Song D, et al. Research on cutting track and working load of directional drilling PDC bit. Journal of Petroleum Science and Engineering, 2022, 208: 109480.

[3] Wang P, Zhao B, Ni H, et al. Research on the modulation mechanism and rock breaking efficiency of a cuttings waterjet. Energy Science & Engineering, 2019, 7(5): 1687–1704.

[4] Zhu B, Xiong L, Xu M. Double-edged cutting simulation with a new combined constitutive model for AISI 1045 steel. Journal of Materials Processing Technology, 2022, 302: 117496.

[5] You Q, Xu M, Zhu B, et al. Experimental Modeling of the Bifurcation Set Equation of the Chip-Splitting Catastrophe in Symmetrical Straight Double-Edged Cutting. Metals, 2022, 12(5): 878.

[6] Liu W, Deng H, Zhu X, et al. The PDC cutter-rock interaction behavior in rock cutting: A review. Geoenergy Science and Engineering, 2023: 212168.

[7] Huang K, Zhou C, Yang Y, et al. Working Load Characteristics of the PDC-Cone Composite Bit under Impact and Scraping. Shock and Vibration, 2020, 2020: 1–9.

[8] Kong C, Wang C, Zhu R, et al. Study on the acceleration of single‐cone PDC composite bit. Mathematical Methods in the Applied Sciences, 2022, 45(7): 3896–3920.

[9] Shimizu Y, Jang S H, Gao W. Design and testing of an optical configuration for multi-dimensional measurement of a diamond cutting tool. Measurement, 2016, 94: 934–941.

[10] Yang Y, Yang Y, Ren H, et al. Research on the working mechanism of the PDC drill bit in compound drilling. Journal of Petroleum Science and Engineering, 2020, 185: 106647.

[11] Zhang C L, Yang Y X, Qi Q L, et al. Research on numerical drilling technology of mesh-like cutting PDC bit. Energy Reports, 2021, 7: 2068–2080.

[12] Gao W, Ge M, Chen D, et al. Back analysis for rock model surrounding underground roadways in coal mine based on black hole algorithm. Engineering with computers, 2016, 32: 675–689.

[13] Mehranpour M H, Kulatilake P H S W, Xingen M, et al. Development of new three-dimensional rock mass strength criteria. Rock Mechanics and Rock Engineering, 2018, 51: 3537–3561.

[14] Zhang Q, Li C, Quan X, et al. New true-triaxial rock strength criteria considering intrinsic material characteristics. Acta Mechanica Sinica, 2018, 34: 130–142.

[15] You M. True-triaxial strength criteria for rock. International Journal of Rock Mechanics and Mining Sciences, 2009, 46(1): 115–127.

[16] Wang C, He B, Hou X, et al. Stress–energy mechanism for rock failure evolution based on damage mechanics in hard rock. Rock Mechanics and Rock Engineering, 2020, 53: 1021–1037.

[17] Zhou P, Li J, Jiang Y, et al. Damage mechanism of tunnels in the high-content salt rock stratum. Bulletin of Engineering Geology and the Environment, 2021, 80: 7633–7652.

[18] Li Y, Zhang T, Tian Z, et al. Simulation on compound percussive drilling: Estimation based on multidimensional impact cutting with a single cutter. Energy Reports, 2021, 7: 3833–3843.

[19] Cheng Y, Zhang X, Lv M, et al. Research on CFRP cutting mechanism by the micro-textured tool using macroscopic and microscopic numerical simulations. Journal of Reinforced Plastics and Composites, 2023, 42(11–12): 577–588.

[20] Zhang X, Huang X, Qi S, et al. Numerical Simulation on Shale Fragmentation by a PDC Cutter Based on the Discrete Element Method. Energies, 2023, 16(2): 965. doi: 10.3390/en16020965

[21] Liu W, Luo Y, Zhu X, et al. The ductile–brittle failure mode transition of hard brittle rock cutting—new insights from numerical simulation. Geomechanics and Geophysics for Geo-Energy and Geo-Resources, 2022, 8(4): 129. doi: 10.1007/s40948-022-00438-7

[22] Sun F, Chen L, Li Y, et al. Research on Fidelity Performance of Coring Bits during Drilling and Cutting in Deep Extreme Environments. Applied Sciences, 2023, 13(14): 8173. doi: 10.3390/app13148173

[23] Yang F, Liu W, Zhu X, et al. The Rock-Breaking Mechanism of Thermal Spalling-Assisted Rock Cutting by PDC Cutter. Rock Mechanics and Rock Engineering, 2023: 1–20.

